# Assessment of preoperative anxiety and depression in patients with pulmonary ground-glass opacities: Risk factors and postoperative outcomes

**DOI:** 10.3389/fsurg.2023.1102352

**Published:** 2023-01-30

**Authors:** Yu Han, Qiduo Yu, Qianli Ma, Jin Zhang, Yuhui Shi, Zhenrong Zhang, Guangliang Qiang, Fei Xiao, Chaoyang Liang

**Affiliations:** ^1^ Department of General Thoracic Surgery, China-Japan Friendship Hospital, Beijing, China; ^2^ Department of Thoracic Surgery, National Center for Respiratory Medicine, Beijing, China

**Keywords:** ground-glass opacity, anxiety, depression, morbidity, quality of life, tumor

## Abstract

**Objective:**

A large number of patients with pulmonary ground-glass opacities (GGOs) have anxiety and depression. However, the contributing factors and effects of anxiety and depression on postoperative outcomes are still unclear.

**Methods:**

Clinical data for patients undergoing surgical resection for pulmonary GGOs were collected. We prospectively evaluated levels and risk factors for anxiety and depression in patients with GGOs before surgery. The relationship between psychological disorders and postoperative morbidity was evaluated. Quality of life (QoL) was also assessed.

**Results:**

A total of 133 patients were enrolled. Prevalence rates of preoperative anxiety and depression were 26.3% (*n* = 35) and 18% (*n* = 24), respectively. Multivariate analysis revealed depression [odds ratio(OR) = 16.27, *p* < 0.001] and multiple GGOs (OR = 3.146, *p* = 0.033) to be risk factors for preoperative anxiety. Anxiety (OR = 52.166, *p* < 0.001), age > 60 (OR = 3.601, *p* = 0.036), and unemployment (OR = 8.248, *p* = 0.006) were identified as risk factors for preoperative depression. Preoperative anxiety and depression were associated with lower QoL and higher postoperative pain scores. Our results also revealed that the incidence of postoperative atrial fibrillation was higher in patients with than in those without anxiety.

**Conclusions:**

In patients with pulmonary GGOs, comprehensive psychological assessment and appropriate management are required before surgery to improve QoL and reduce postoperative morbidity.

## Introduction

Over the past decades, low-dose computed tomography (LDCT) has been widely applied in lung cancer screening for high-risk patients (1). As a result, an increasing number of pulmonary ground glass opacities (GGOs) have been identified (2, 3). It has been proven that pulmonary nodules manifest as GGOs that enlarge slowly and are associated with excellent survival after resection (4, 5). However, due to the fear of being diagnosed with lung cancer and undergoing surgery, a large number of patients have psychological disorders, most commonly anxiety and depression (6, 7).

Previous studies have shown that 20.9-65.0% of patients with lung cancer have anxiety symptoms, and the incidence of depression ranges from 38.9%–65% (8–11). The prevalence of anxiety and depression in lung cancer patients is influenced by a variety of factors, including age, sex, social support, comorbidities, tumor stage and other factors (12–14). Until now, however, the psychological status and risk factors for anxiety and depression in patients with pulmonary GGOs who have not been diagnosed with lung cancer have remained unclear.

It has been revealed that anxiety and depression are associated with lower QoL in patients with lung cancer (15, 16). Preoperative anxiety has also been proven to be related to higher morbidity and mortality after cardiovascular surgery (17). Furthermore, depressive emotion has even been associated with worse survival in patients with lung cancer (18). Nevertheless, the association of preoperative anxiety and depression with postoperative outcomes of pulmonary resection is still unclear.

In this article, we describe psychological assessments of patients with pulmonary GGOs before surgery, the QoL of those patients, and the occurrence of postoperative outcomes. We aimed to investigate the prevalence of and contributing factors for preoperative psychological disorders in patients with pulmonary GGOs and whether anxiety and depression have adverse effects on QoL and postoperative outcomes in these patients.

## Methods

### Patient population

**Figure 1:**
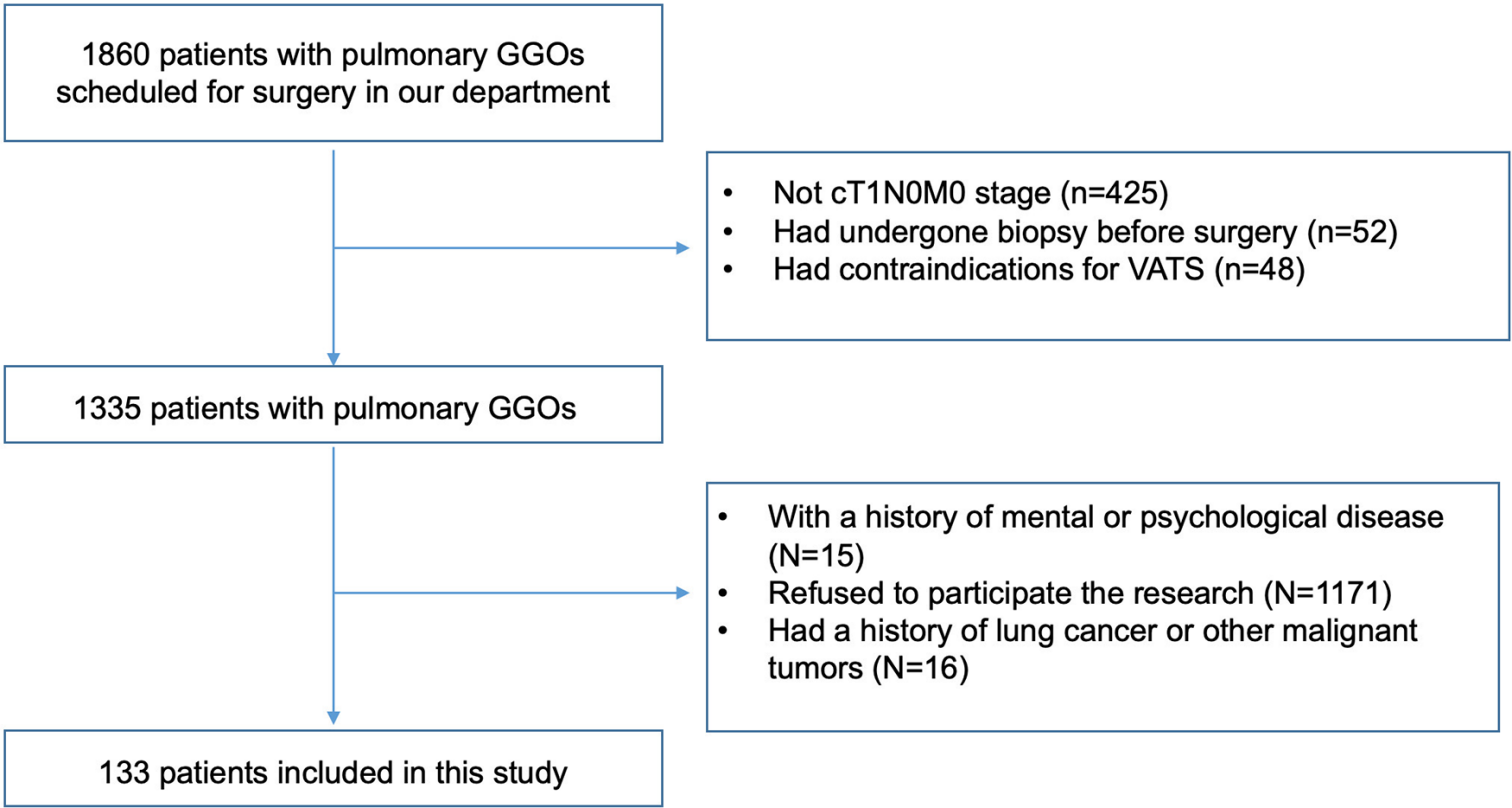
Flow chart of the study.

From October 2020 to August 2022, 133 patients who underwent thoracoscopic pulmonary resection in China-Japan Friendship Hospital for pulmonary GGOs were included in this study (Figure 1). The patients selected for this research met the following criteria: (1) pulmonary GGOs suspected to be malignant with indications for minimally invasive surgery; (2) clinical stages judged to be cT1N0M0 according to the 8th edition of the AJCC TNM staging system; and (3) not undergoing pathological biopsy before surgery. The exclusion criteria were as follows: (1) a history of mental or psychological diseases; (2) a history of lung cancer or other malignant tumors; and (3) refusal to participate in the study. Patient-controlled intravenous analgesia (PCIA) combined with intercostal nerve block was used for postoperative analgesia. Written informed consent was obtained from all patients before surgery. This study was approved by our institutional review board (2022-KY-127).

### Measurement instruments

All patients received psychological evaluations with the Hospital Anxiety Depression Scale (HADS) questionnaire during hospitalization before surgery. The HADS is a widely utilized self-report questionnaire designed to screen for anxiety and depression states in patients (19). The questionnaire consists of the HADS-A and HADS-D, which are designed to detect anxious and depressive states, respectively. Each subscale contains seven items, and each question is scored from 0 to 3 points. Higher scores represent higher levels of anxious or depressive states, and the total scores can range from 0 to 21 in each subgroup. HADS scores ≥8 were defined as anxiety or depression in this study (20).

QoL was assessed with the EORTC QLQ-C30 questionnaire (21), which is the most widely utilized cancer-specific Health-Related Quality-of-Life instrument. The EORTC QLQ-C30 consists of five functional dimensions on physical, role, emotional, cognitive and social functioning, three symptom items (pain, nausea/vomiting, fatigue), six single items including dyspnea, insomnia, appetite loss, constipation, diarrhea, and financial impact, and a global health scale. The scoring procedures were performed as previously described (22).

### Statistical methods

Statistical analysis was performed using SPSS (version 23, IBM Inc., Chicago, IL, USA). Categorical variables were compared using the chi-square test or Fisher's exact test. Student's *t test* or the Wilcoxon rank-sum test was used to analyze continuous variables. A *p* value less than 0.05 was considered statistically significant. A binary logistic regression test was performed to detect risk factors for anxiety and depression status. Variables with a *p* value less than 0.15 in univariate analysis were then included in multivariate analysis. A *p* value less than 0.05 was regarded as statistically significant for both univariate and multivariate analyses.

## Results

### Basic patient characteristics and perioperative outcomes

The basic patient characteristics are shown in Table 1. A total of 133 patients with pulmonary GGOs met the inclusion criteria. The case series consisted of 41 (30.8%) men and 92 (69.2%) women, with an average age of 51.5 ± 11.7 years. Sixty-four patients (48.1%) had low levels of education (middle school and below). One hundred and five patients (78.9%) were married, and 70 patients (52.6%) were employed. Twenty-seven patients (20.3%) had a history of alcohol consumption, and 14 patients (10.5%) were smokers. Hypertension was the most common comorbidity (21.1%) in this case series; other common comorbidities included diabetes mellitus (9.0%), pulmonary diseases (6.0%), and cardiac diseases (5.3%). Most of the nodules were malignant (87.2%), and 98.5% were stage I. Overall postoperative morbidity was 15.8%, and no patient died within 30 days postoperatively. The median postoperative length of hospitalization (LOH) was 4 days (IQR: 3–4 days).

**Table 1:** Basic patient characteristics and perioperative outcomes.

Parameters	Values
Age, years	51.5 ± 11.7
Male	41 (30.8)
Social status	
Low levels of education	64 (48.1)
Currently married	105 (78.9)
Currently employed	70 (52.6)
Alcohol consumption	27 (20.3)
History of smoking	14 (10.5)
Comorbidity	43 (32.3)
Hypertension	28 (21.1)
Diabetes mellitus	12 (9.0)
Pulmonary disease	8 (6.0)
Cardiac disease	7 (5.3)
Cerebrovascular disease	2 (1.5)
Patient's psycho-emotional status	
Patients with anxiety	35 (26.3)
Patients with depression	24 (18.0)
Postoperative outcomes	
30-day morbidity	21 (15.8)
Atrial fibrillation	6 (4.5)
Air leak	4 (3.0)
Pulmonary infection	9 (6.8)
Pulmonary Embolism	1 (0.8)
Wound infection	1 (0.8)
30-day mortality	0
Length of stay, days	4 (3-4)
Tumor size, mm	12.9 ± 6.1
Pathology	
Benign	17 (12.8)
Malignant	116 (87.2)
Pathological stage	
Stage I	131 (98.5)
Stage II	1 (0.8)
Stage III	1 (0.8)

### Prevalence of preoperative anxiety and depression

Based on HADS scores, 41 patients (30.8%) had anxiety or depression. Thirty-five patients (26.3%) had anxiety, and 24 patients (18.0%) had depression before surgery. Moreover, 18 patients (13.5%) were identified as having from both anxiety and depression. All patients with HADS scores ≥8 were recommended to receive psychiatric counselling before surgery. However, only 12 patients (29.3%) agreed to visit the mental health clinic. Among those patients, 6 (50.0%) were diagnosed with adjustment disorder, 4 (33.3%) with anxiety disorder and 2 (16.7%) with depression disorder, and supportive psychotherapy or psychiatric medication was provided.

### Risk factors for preoperative anxiety and depression

We further analyzed risk factors for preoperative anxiety and depression (Table 2). Patients with multiple GGOs were more likely to have anxiety than those with a single lesion (*p* = 0.010). Moreover, preoperative depression was another risk factor associated with anxiety (*p* < 0.001). Conversely, no significant differences between the two groups were observed in terms of sex, age, education level, marital status, employment status, history of chronic disease, or GGO image size (*p* > 0.05). After univariable analysis, sex, number of lesions, and preoperative depression were qualified for multivariable analysis. Based on multivariable analysis, preoperative depression (*p* < 0.001) and the number of lesions (*p* = 0.033) were identified as risk factors for preoperative anxiety (Table 3).

**Table 2:** Analysis of risk factors of anxiety and depression for patients with pulmonary GGO.

Parameters	Anxiety		*p*-value	Depression		*p*-value
	Yes (*n* = 35)	No (*n* = 98)		Yes (*n* = 24)	No (*n* = 109)	
Gender			0.106			0.242
Male	7 (20.0)	34 (34.7)		5 (20.8)	36 (33.0)	
Female	28 (80.0)	64 (65.3)		19 (79.2)	73 (66.9)	
Age, years			0.664			0.040
<40	5 (14.3)	14 (14.3)		2 (8.3)	17 (15.6)	* *
40-60	23 (65.7)	57 (58.2)		11 (45.8)	69 (63.3)	* *
>60	7 (20.0)	27 (27.6)		11 (45.8)	23 (21.1)	* *
Education levels			0.482		*** ***	0.047
Elementary school and below	3 (8.6)	5 (5.1)		4 (16.7)	4 (3.7)	
Middle school	12 (34.3)	44 (44.9)		10 (41.7)	46 (42.2)	
College and undergraduate	20 (57.1)	49 (50.0)		10 (41.7)	59 (54.1)	
Marital status			0.859			0.977
Married	28 (80.0)	77 (78.6)		19 (79.2)	86 (78.9)	
Unmarried or Divorced or widowed	7 (20.0)	21 (21.4)		5 (20.8)	23 (21.1)	
Currently employed			0.868			0.001
Yes	18 (51.4)	52 (53.1)		5 (20.8)	65 (59.6)	
No	17 (48.6)	46 (46.9)		19 (79.2)	44 (40.4)	
History of chronic disease			0.894			0.041
Yes	11 (31.4)	32 (32.7)		12 (50.0)	31 (28.4)	
No	24 (68.6)	66 (67.3)		12 (50.0)	78 (71.6)	
HADS						
Anxiety Scale	9 (8–11)	3 (2–6)	<0.001	9 (7.25–11)	4 (2–6)	<0.001
Depression Scale	8.5 (5–10)	2 (1–4)	<0.001	9.5 (8–11)	2 (1–3.5)	<0.001
Imaging size of GGO, mm	12.8 ± 6.2	12.9 ± 6.1	0.950	14.4 ± 6.7	12.6 ± 5.9	0.186
Components of GGO			0.585			0.214
Pure GGO	9 (25.7)	30 (30.6)		4 (16.7)	35 (32.1)	
Mixed GGO	26 (74.3)	68 (69.4)		20 (83.8)	74 (67.9)	
Number of lesion		*** ***	0.010			0.189
Single GGO	23 (65.7)	84 (85.7)		17 (70.8)	90 (82.6)	
Multiple GGOs	12 (34.3)	14 (14.3)		7 (29.2)	19 (17.4)	
Pathology			0.384			0.737
Benign	6 (17.1)	11 (11.2)		2 (8.3)	15 (13.8)	
Malignant	29 (82.9)	87 (88.8)		22 (91.7)	94 (86.2)	

GGO, ground-glass opacity; HADS, Hospital Anxiety Depression Scale.

**Table 3:** Univariable and multivariable risk factor analyses for anxiety.

Variables	Univariable analysis			Multivariable analysis		
	OR	95% Cl	*p*-value	OR	95% Cl	*p*-value
Female	2.125	0.841–5.369	0.106			
Depression disorder	16.235	5.631–46.812	<0.001	16.270	5.491–48.212	<0.001
Multiple GGOs	3.130	1.275–7.688	0.010	3.146	1.100–8.995	0.033

Cl, confidence interval; OR, odds ratio.

In univariate analysis, the variables related to preoperative depression were anxiety disorder (*p* < 0.001), age > 60 (*p* = 0.008), low education level (*p* = 0.068), current unemployment (*p* = 0.001), and history of chronic diseases (*p* = 0.041). Furthermore, anxiety disorder (*p* < 0.001), age > 60 (*p* = 0.036), and current unemployment (*p* = 0.006) were significantly associated with preoperative depression in multivariate analysis (Table 4).

**Table 4:** Univariable and multivariable risk factor analyses for depression.

Variables	Univariable analysis			Multivariable analysis		
	OR	95% Cl	*p*-value	OR	95% Cl	*p*-value
Anxiety disorder	16.235	5.631–46.812	<0.001	52.166	10.044–270.945	<0.001
Age >60, years	2.889	1.325–6.299	0.008	3.601	1.087–11.932	0.036
Low education level	1.929	0.953–3.905	0.068			
Currently unemployed	5.614	1.951–16.152	0.001	8.248	1.844–36.880	0.006
History of chronic disease	2.516	1.021–6.200	0.041			

Cl, Confidence interval; OR, Odds ratio.

### Quality of life of patients with pulmonary GGOs before surgery

Compared with those without anxiety or depression, patients with anxiety and depression had lower scores on all aspects of QoL (physical, role, emotional, cognitive, and social functioning) and global health. There were significant differences between the anxiety and nonanxiety groups regarding insomnia (*p* = 0.015) and appetite loss (*p* = 0.012). Additionally, patients with preoperative depression had lower scores of QoL in terms of fatigue (*p* = 0.021), pain (*p* = 0.033), insomnia (*p* = 0.039) and appetite loss (*p* = 0.006). Detailed score reports of the EORTC QLQ-30 are shown in Table 5.

**Table 5:** Quality of life of the patients with pulmonary GGO before surgery.

Parameters	Anxiety		*p*-value	Depression		*p*- value
	Yes (*n* = 35)	No (*n* = 98)		Yes (*n* = 24)	No (*n* = 109)	
Global health (status/QoL)	66.7(50.0–83.3)	83.3 (66.7–91.7)	0.004	58.3 (41.7–83.3)	83.3 (66.7–91.7)	<0.001
Physical functioning	93.3 (80–100)	100 (93.3–100)	0.019	90 (73.3–100.0)	100 (93.3–100.0)	0.006
Role functioning	100 (66.7–100)	100 (100–100)	<0.001	66.7 (54.2–100.0)	100 (100–100)	<0.001
Emotional functioning	66.7 (50.0–91.7)	91.7 (75.0–100.0)	<0.001	66.7 (50–97.9)	91.7 (66.7–100.0)	0.005
Cognitive functioning	83.3 (66.7–100.0)	100 (83.3–100.0)	0.011	66.7 (50.0–100.0)	100 (83.0–100.0)	<0.001
Social functioning	83.3 (66.7–100.0)	100 (83.3–100)	0.015	83.3 (66.7–100)	100 (83.3–100.0)	<0.001
Fatigue	22.2 (0–33.3)	11.1 (0–22.2)	0.160	22.2 (2.8–33.3)	11.1 (0–22.2)	0.021
Nausea and vomiting	0 (0–0)	0 (0–0)	0.133	0 (0–0)	0 (0–0)	0.382
Pain	0 (0–16.7)	0 (0–16.7)	0.505	16.7 (0–16.7)	0 (0–16.7)	0.033
Dyspnea	0 (0–33.3)	0 (0–33.3)	0.500	16.7 (0–33.3)	0 (0–33.3)	0.239
Insomnia	33.3 (0–66.7)	0 (0–33.3)	0.015	33.3 (0–66.7)	0 (0–33.3)	0.039
Appetite loss	0 (0–33.3)	0 (0–33.3)	0.012	33.3 (0–33.3)	0 (0–33.3)	0.006
Constipation	0 (0–33.3)	0 (0–33.3)	0.644	0 (0–25)	0 (0–0)	0.306
Diarrhea	0 (0–0)	0 (0–0)	0.309	0 (0–0)	0 (0–0)	0.081
Financial difficulties	0 (0–33.3)	0 (0–33.3)	0.450	0 (0–33.3)	0 (0–0)	0.538

QoL, quality of life.

### Effects of preoperative anxiety and depression on short-term postoperative outcomes

The surgical approaches were comparable among the different groups (Table 6). We further investigated the association of preoperative anxiety and depression with postoperative outcomes among different subgroups (Table 6). Pain scores were significantly higher in the anxiety group at postoperative Day 1 (5 [interquartile range (IQR), 4–6] vs. 4 [IQR, 3–5], *p* < 0.001), POD 2 (3 [IQR, 3–4] vs. 2 [IQR, 2–3], *p* < 0.001), and POD 3 (2 [IQR, 1–2] vs. 1.5 [IQR, 1–2], *p* = 0.012). The incidence of atrial fibrillation was also higher in patients with preoperative anxiety than in those without anxiety (11.4% vs. 2.0%, *p* = 0.041). However, other postoperative outcomes were comparable between the two groups, as were chest tube duration (*p* = 0.412) and length of stay (*p* = 1.000).

**Table 6:** Effects of anxiety and depression on postoperative outcomes.

Parameters	Anxiety		*p*-value	Depression		*p*-value
	Yes (*n* = 35)	No (*n* = 98)		Yes (*n* = 24)	No (*n* = 109)	
Pain scores
POD 1	5 (4–6)	3 (3–5)	<0.001	4.5 (4–6)	4 (3–5)	0.009
POD 2	3 (3–4)	2 (2–3)	<0.001	3 (3–3.8)	2 (2–3)	0.003
POD 3	2 (1–2)	1.5 (1–2)	0.012	2 (1.3–2)	2 (1–2)	0.061
Surgical approach			0.306			0.901
Wedge resection	6 (17.1)	30 (30.6)		6 (25)	30 (27.5)	
Segmentectomy	15 (42.9)	35 (35.7)		10 (41.7)	40 (36.7)	
Lobectomy	14 (40.0)	33 (33.7)		8 (33.3)	39 (35.8)	
30-day morbidity	7 (20.0)	14 (14.3)	0.426	3 (12.5)	18 (16.5)	0.765
Atrial fibrillation	4 (11.4)	2 (2.0)	0.041	2 (8.3)	4 (3.7)	0.296
Air leak	0	4 (4.1)	0.573	0	4 (3.7)	1.000
Pulmonary infection	3 (8.6)	6 (6.1)	0.698	1 (4.2)	8 (7.3)	1.000
Pulmonary Embolism	0	1 (1.0)	1.000	0	1 (0.9)	1.000
Wound infection	0	1 (1.0)	1.000	0	1 (0.9)	1.000
Chest tube duration, days	3 (2–3)	3 (2–3)	0.412	3 (2–3)	3 (2–3)	0.704
Length of stay, days	4 (3–4)	4 (3–4)	1.000	4 (3–4)	4 (3–4)	0.848

POD, postoperative day.

Postoperative pain at POD 1 (4.5 [IQR, 4–6] vs. 4 [IQR, 3–5], *p* = 0.009) and POD 2 (3 [IQR, 3–3.8] vs. 2 [IQR, 2–3], *p* = 0.003) was also significantly higher in patients with than in those without depression. However, no significant differences were observed between the depression and non-depression groups regarding other postoperative outcomes, including chest tube duration (*p* = 0.704) and length of stay (*p* = 0.848).

## Discussion

With the wide implementation of lung cancer screening, an increasing number of early-stage NSCLC cases have been identified, usually manifesting as GGOs (3, 4). However, few studies have focused on the psychologic status of these patients. This study aimed to elucidate the prevalence and risk factors for anxiety and depression in patients with pulmonary GGOs. QoL and postoperative outcomes were also investigated in our research.

Based on our evaluation, a large number of patients with GGOs have anxiety or depression disorder (30.8%). Moreover, the occurrence rate of anxiety (26.3%) was higher than that of depression (18.0%). Li and colleagues (13) also revealed that 31.8% of patients with incidental pulmonary nodules have anxiety and that 19.4% have depression disorder. These findings indicate that anxiety and depression widely exist in patients with pulmonary GGOs and should not be ignored.

In our research, the number of GGOs was identified as a risk factor for preoperative anxiety. Compared to a single lesion, the treatment strategy for multiple GGOs is more complex and difficult, which may cause nervousness and anxiety. Anxiety and depression are also linked to social-economical factors and support from the institutions (23). We demonstrated that low levels of education and current unemployment are risk factors for depression. Older patients (>60 years) were also more vulnerable to depression than younger patients based on our research.

We found that preoperative depression is an independent risk factor for anxiety disorder and that preoperative anxiety is a risk factor for depression. Comorbidities of anxiety and depression in patients with lung cancer have been found in other studies (24, 25). Furthermore, preoperative anxiety and depression were associated with worse QoL in the patients with GGOs in our study. Hence, to improve QoL and postoperative outcomes, it is important to identify patients with anxiety or depression preoperatively and to offer professional psychological counselling and management.

Acute postoperative pain is an important problem after thoracic surgery and may increase pulmonary and cardiac complications and decrease quality of life. De Cosmo et al. found that patients with preoperative anxiety and depression had higher pain intensities after laparoscopic cholecystectomy (26). Our study revealed that preoperative anxiety and depression both significantly increased postoperative pain in patients who underwent thoracoscopic pulmonary resection. Therefore, special attention should be given to whether such patients experience severe postoperative pain, and adequate analgesics should be administered in a timely manner.

Preoperative anxiety and depression disorders have been considered to be strongly associated with extended LOS and increased complications after complex surgery, including colectomy, total hip arthroplasty, and lung resection (27). Our study found that the incidence of postoperative atrial fibrillation (AF) was higher in patients with preoperative anxiety. Anxiety may cause postoperative AF by increasing sympathetic tone, as based on other studies (28).

Several limitations of our study should be considered. First, postoperative psychological disorders were not evaluated, which may have changed when the patients were informed of the pathology. A dynamic evaluation of anxiety and depression would be more helpful to understand the psychological state of patients. Second, although patients with anxiety and depression were identified in our study, few of them followed advice for psychological counseling or therapy. Third, the surgical approaches had been planned and discussed with patients before admission in our study, patients who scheduled for lobectomy may suffer higher levels of anxiety or depression than patients who scheduled for wedge resection due to different risks of surgery. Furthermore, because anxiety and depression are perceived differently according to the patients beliefs and country of origin. All the patients came from China in our study. We think patients with different beliefs and countries should be included in further studies so that the conclusions in our study could be generalized. Finally, further studies are needed to eliminate preoperative anxiety and depression in patients with GGOs by popularizing medical knowledge and cooperating with professional psychologists to provide multidisciplinary care.

## Conclusion

In conclusion, anxiety and depression are common psychological disorders among patients with pulmonary GGOs. Based on our study, preoperative anxiety and depression are related to lower QoL, severe postoperative pain, and a higher occurrence of postoperative AF. Psychological assessment and appropriate management are required for patients with GGOs who have anxiety or depression.

## Data Availability

The raw data supporting the conclusions of this article will be made available by the authors, without undue reservation.
